# Antioxidative and Antimycotoxigenic Efficacies of *Thunbergia laurifolia* Lindl. for Addressing Aflatoxicosis in Cherry Valley Ducks

**DOI:** 10.3390/toxins16080334

**Published:** 2024-07-27

**Authors:** Chompunut Lumsangkul, Phruedrada Kaewtui, Kiattisak Huanhong, Ko-Hua Tso

**Affiliations:** 1 Department of Animal and Aquatic Sciences, Faculty of Agriculture, Chiang Mai University, Chiang Mai 50200, Thailand; phruedrada_k@cmu.ac.th (P.K.); kiattisak_huanhong@cmu.ac.th (K.H.); 2 Multidisciplinary Research Institute, Chiang Mai University, 239 Huay Keaw Rd., Chiang Mai 50200, Thailand; 3 Department of Animal Science, National Chung Hsing University, Taichung 40227, Taiwan; 4 Dr. Bata Ltd., Bajcsy-Zs. u. 139, H-2364 Ócsa, Hungary

**Keywords:** antimycotoxigenic, aflatoxin B_1_, cherry valley ducks, *Thunbergia laurifolia* extract

## Abstract

This study aimed to assess the effectiveness of aflatoxin B_1_ (AFB_1_) and *Thunbergia laurifolia* extract (TLE) in the diets of Cherry Valley ducklings. Our investigation covered growth indicators, blood biochemical indices, meat quality, intestinal morphology, immune response, and CP450 enzyme-related gene expression. We conducted the study with 180 seven-day-old Cherry Valley ducks, randomly divided into five dietary treatments. These treatments included a basal diet without AFB_1_ (T1 group), TLE, or a commercial binder; the basal diet containing 0.1 mg AFB_1_/kg (T2 group), 0.1 mg AFB_1_/kg and 100 mg TLE/kg (T3 group), 0.1 mg AFB_1_/kg and 200 mg TLE/kg (T4 group), and 0.1 mg AFB_1_/kg and 0.5 g/kg of a commercial binder (T5 group), respectively. Ducklings fed with the T2 diet exhibited lower final body weight (BW), average body weight gain (ADG), and poor feed conversion ratio (FCR) during the 42-day trials. However, all ducklings in the T3, T4, and T5 groups showed significant improvements in final BW, ADG, and FCR compared to the T2 group. Increased alanine transaminase (ALT) concentration and increased expression of CYP1A1 and CYP1A2 indicated hepatotoxicity in ducklings fed the T2 diet. In contrast, ducklings fed T3, T4, and T5 diets all showed a decrease in the expression of CYP1A1 and CYP1A2, but only the T4 treatment group showed improvement in ALT concentration. AFB_1_ toxicity considerably raised the crypt depth (CD) in both the duodenum and jejunum of the T2 group, while the administration of 200 mg TLE/kg (T4) or a commercial binder (T5) effectively reduced this toxicity. Additionally, the villus width of the jejunum in the T2 treatment group decreased significantly, while all T3, T4, and T5 groups showed improvement in this regard. In summary, *T. laurifolia* extract can detoxify aflatoxicosis, leading to growth reduction and hepatic toxicosis in Cherry Valley ducklings.

## 1. Introduction

Aflatoxins are secondary fungal metabolites, or mycotoxins, primarily produced by toxigenic strains of the fungi *Aspergillus flavus* and *Aspergillus parasiticus* [1]. These mycotoxins are classified as carcinogenic furanocoumarins and consist of twenty related polycyclic structures [2]. Aflatoxin B_1_ (AFB_1_), the most toxic and prevalent aflatoxin, causes oxidative stress, leading to severe hepatoxicity. It also inhibits growth and reproductive performance in poultry, resulting in significant negative effects on animal health, food security, and economic trade [3,4,5]. Aflatoxins pose a particular problem in hot and dry climates that favor mycotoxigenic fungal growth. Therefore, one of the most severely contaminated areas of AFB_1_ in the world is Southeast Asia, especially Thailand, which often experiences higher levels of contamination [6]. Previous research indicated that 38.9% of 3206 samples were highly contaminated with aflatoxin, and the prevalence of aflatoxin reached 44.3% in local corn samples [7].

Poultry aflatoxicosis, traced back to the 1960 outbreak of turkey X diseases in the UK, remains a significant threat to the global poultry industry today [8]. Aflatoxin-contaminated feeds, exacerbated by climate change, continue to cause poor growth performance, compromised reproductive ability, liver necrosis, and bile duct hyperplasia in poultry, leading to substantial economic losses [9]. The detrimental impact extends to bone metabolism, resulting in a weakened skeletal structure and decreased meat yield [10]. Among poultry species, ducklings exhibit the highest sensitivity to AFB_1_ [11,12] because waterfowls have high levels of unsaturated fatty acids in their body tissues, making them more susceptible to lipid peroxidation induced by AFB_1_ [13,14,15]. For ducklings, the mortality rates reached 100% at 1 mg/kg AFB_1_ [10]. Public health concerns arise from aflatoxin residues in poultry products (e.g., eggs and meat), posing risks ranging from mild liver issues to carcinogenesis in consumers [16]. These challenges underscore the urgent need for stringent regulations and effective mitigation strategies to safeguard poultry welfare and human health while preserving the economic viability of the poultry industry. 

The physical characteristics of aflatoxins include high heat stability and polarity [17]. Hence, the efficacy of detoxifying AFB_1_ via thermal inactivation is relatively limited. On the other hand, because of the high polarity of aflatoxins, binders exhibit high adsorption ability, making binder supplements the main detoxifying strategy of AFBs in current farms and feed mills [18]. However, binders not only remove AFB_1_ but also absorb some nutrition compounds (e.g., zinc and vitamin B group) in feed [19]. The long-term addition of high amounts of adsorbents can cause zinc deficiency, leading to poultry being unable to stand [20]. Phytobiotic feed additives with antioxidant functions appear to be a good choice for detoxifying AFB_1_ in poultry. When the feed contains high levels of AFB_1_, phytobiotic feed additives with antioxidant functions can neutralize the mycotoxin toxicities for poultry. When the content is low, they can have multiple uses (e.g., improving immunity and growth traits) for birds [21,22]. 

*Thunbergia laurifolia* (Rang chuet) extract (TLE) is widely used for neutralizing toxicities from various toxins [23,24]. It is also a common antidote for several poisonous agents in Thai traditional medicine [25,26]. In addition, TLE contains phenolic compounds, which are involved in anti-inflammation and antioxidants [27,28]. Several papers have reported that apigenin, one of the flavonoid compounds in TLE [29], has antioxidant [30] and anticancer properties [31]. The main toxicity of AFB_1_ is oxidative stress occurrence via reactive oxygen species production [32]. We hypothesized that TLE had the potential to inhibit aflatoxicosis through its antioxidant ability. In addition, there is no available data about the effects of these herbal medicine products on the duck. Therefore, the poultry industry should develop alternative strategies for detoxifying mycotoxins by TLE in ducks. Hence, the objectives of this research are to comprehensively assess the effects of AFB_1_ along with TLE as a natural feed additive in duckling diet on the growth performance, serum biochemical parameters, intestine morphology, carcass traits, meat quality, and immunity responses of Cherry Vally ducks.

## 2. Results

### 2.1. Phytochemical and Antioxidant Activity of T. laurifolia Extracts

The results showed that the total phenol compound was 0.56 mg GAE/g, and the antioxidant activity, as indicated by the values of 2,2-diphenyl-1-picrylhydrazyl radical (DPPH), 2,2-azino-bis (3-ethylbenzothiazo-line-6-sulfonic acid) (ABTS), and ferric reducing antioxidant power (FRAP), amounted to 7.26 μmol Trolox equivalents (TE)/g, 3.70, and 51.26 mM Fe^2+^/g, respectively (Table 1). 

### 2.2. Growth Performance

The average daily gain (ADG), the average daily feed intake (ADFI), and the feed conversion ratio (FCR) are presented in Table 2. The final body weight (BW) and ADG were significantly (*p* < 0.05) reduced by AFB_1_ during the growth phase (7 to 42 days). However, feeding *T. laurifolia* extract and commercial mycotoxin binder along with AFB_1_ significantly improved ADG during days 7–42. There was no significant difference in ADFI between the groups. The FCR during 7 to 42 days was significantly (*p* < 0.05) higher in the AFB_1_-challenged groups. Nevertheless, feeding *T. laurifolia* extract and a commercial mycotoxin binder significantly enhanced the FCR compared to AFB_1_-fed birds, and it was comparable to that of ducks in the control group.

### 2.3. Blood Biochemistry

Aflatoxin B_1_ exhibited significant toxic effects by significantly increasing (*p* < 0.05) the levels of total cholesterol, triglyceride, aspartate transaminase (AST), and globulin (Table 3) in serum biochemical values. When the AFB_1_-contaminated diet was supplemented with 100 and 200 mg/kg of TLE or 0.5 g/kg of commercial binder, lower concentrations of AST were observed in the serum of the ducklings compared to those fed without these detoxifying agents (*p* < 0.001). Additionally, the AST values in ducklings fed the AFB_1_-contaminated diet with TLE treatment were significantly reduced compared to those of the commercial binder treatment.

### 2.4. Intestine Morphology

Aflatoxin B_1_ had significantly unequal effects on the different parts of the examined intestine morphology (*p* < 0.01, Table 4). In general, the villus height (VH) of the duodenum, jejunum, and ileum in ducklings fed the diet containing 0.1 mg/kg AFB_1_ was higher than those of ducklings fed the control diet. However, all detoxifying treatments did not decrease the VH but rather increased the values. Notably, AFB_1_ increased crypt depth (CD) in the duodenum and jejunum (*p* < 0.0001) but reduced CD in the ileum (*p* = 0.0011), while those fed with the AFB_1_-contaminated diet supplemented with 200 mg/kg TLE improved these phenomena. The toxicity of AFB_1_ yielded contrasting results in the villus width (VW) of the duodenum and jejunum. Compared to the control group, the VW in the duodenum of the AFB_1_-contaminated group was higher (*p* = 0.0015), while the VW in the jejunum of the AFB_1_-contaminated group was lower (*p* = 0.0028). Additionally, 200 mg/kg TLE ameliorated AFB_1_ toxicity in VW of both parts of the intestine. As for villus height per crypt depth ratio (VH:CD), only the ileum was affected by AFB_1_ (*p* = 0.013), while the treatments of 100 mg/kg TLE and 0.5 g/kg commercial binder treatments rather increased the ratio.

**Figure 1:**
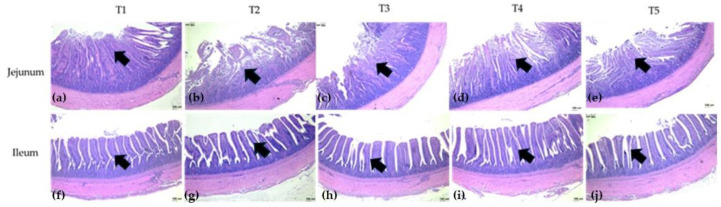
Histological representations of the H&E-stained jejunum and ileum sections of ducks. (**a**) T1: Control, only basal diet without AFB_1_, TLE, or commercial binder, which showed normal histology of intestinal villi with normal pseudostratified epithelium with goblet cells (arrow) in jejunum; (**b**) T2: the basal diet containing 0.1 mg AFB_1_/kg, which AFB_1_ showed significant mucosal necrosis and decreased villi integrity in the jejunum (arrow); (**c**) T3: the basal diet containing 0.1 mg AFB_1_/kg and 100 mg TLE/kg, which showed mild mucosal necrosis and loose villi integrity in the jejunum (arrow); (**d**) T4: the basal diet containing 0.1 mg AFB_1_/kg and 200 mg TLE/kg, which showed slight mucosal necrosis and loose villi integrity in the jejunum (arrow); (**e**) T5: the basal diet containing 0.1 mg AFB_1_/kg and 0.5 g/kg of commercial binder, which showed slight mucosal necrosis and loose villi integrity in the jejunum (arrow). (**f**) T1 showed the complete and compact villus tissue in the ileum (arrow); (**g**) T2 showed loose villus structure in the ileum (arrow); (**h**) T3 showed slightly loose villus structure in the ileum (arrow); (**i**) T4 showed slightly loose villus structure in the ileum (arrow); (**j**) T5 showed slightly loose villus structure in the ileum (arrow); Magnification was 10× the objective lens. Scale bars represent 100 µm.

Light microscopy micrographs of the intestine of each experimental group were shown in Figure 1. It was observed that the photomicrograph of the jejunum sections of the control group (T1) showed normal histology of intestinal villi with normal pseudostratified epithelium with goblet cells. In contrast, the addition of 0.1 mg/kg AFB_1_ had a significant effect on jejunum tissue histopathology. The photomicrograph of the jejunum section of the T2 group (0.1 mg/kg AFB_1_) showed mucosal necrosis. Meanwhile, the photomicrograph of the jejunum section of the T3, T4, and T5 groups (AFB_1_ with TLE or commercial binder) showed a marked improvement in mucosal necrosis with an increase in villi integrity, especially in T4 (0.1 mg AFB_1_/kg and 200 mg TLE/kg) and T5 (0.1 mg AFB_1_/kg and 0.5 g/kg of commercial binder). There were similar results in the ileum sections. The T1 group had relatively complete and compact villus tissue. The T2 group had a looser villus structure than the T1 group due to aflatoxicosis in the ileum villus structure. The T4 and T5 groups had the effect of improving AFB_1_ toxicity.

### 2.5. Carcass Trait, Relative Organ Weight, and Meat Quality

The *T. laurifolia* extract and AFB_1_ supplementation did not influence the relative weight of carcass (excluding neck and feet), breast meat, bursa of Fabricius, or spleen, but there was a tendency for an increase (*p* < 0.1) in liver and gizzard weight. The relative weight of the bursa of Fabricius, spleen, breast meat, and carcass (excluding the neck and feet) was not affected by the *T. laurifolia* extract or AFB_1_ supplementation; however, there was a tendency for the liver and gizzard weight to increase (*p* < 0.1) in duckling fed with T2 and T3 (Table 5). Dietary treatments did not affect the pH test for 45 min and 24 h, thiobarbituric acid reactive substances (TBARS), lightness (L), redness (a), or drip loss (Table 6). However, the inclusion of AFB_1_ increased (*p* < 0.05) shear force and breast meat yellowness (b).

### 2.6. Expression of Immune Response and Metabolizing Cytochrome P450 Enzyme-Related Genes

**Figure 2:**
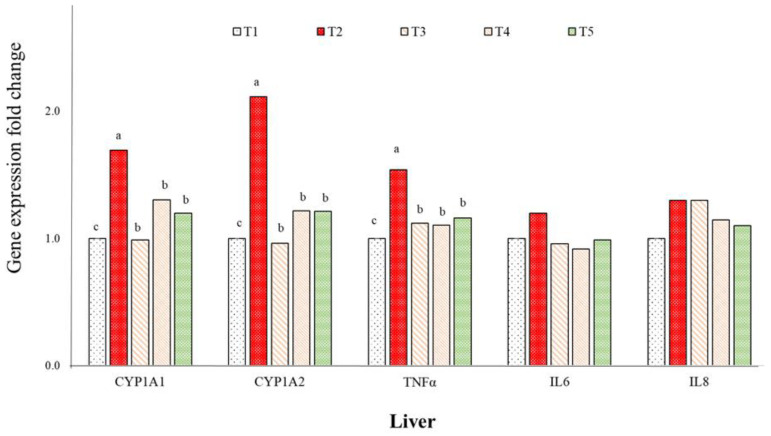
Expressions of immune (tumor necrosis factorα, TNFα; interleukin 6, IL6; interleukin 8, IL8) and metabolizing Cytochrome P450 enzyme (CYP1A1 = cytochrome P450 1A1; CYP1A2 = cytochrome P450 1A2) related genes in liver of aflatoxin-challenged ducks. Three replicates. ^a–c^ Columns without the same superscripts differ (*p* < 0.05). T1: Control, only basal diet without AFB_1_, TLE, or commercial binder; T2: the basal diet containing 0.1 mg AFB_1_/kg; T3: the basal diet containing 0.1 mg AFB_1_/kg and 100 mg TLE/kg; T4: the basal diet containing 0.1 mg AFB_1_/kg and 200 mg of TLE/kg; T5: the basal diet containing 0.1 mg AFB_1_/kg and 0.5 g/kg of commercial binder; AFB_1_: aflatoxin B_1_; TLE: *T. laurifolia* extract.

*T. laurifolia* extract mitigated liver pathological damage caused by AFB_1_ in ducklings. The mRNA levels of the inflammation-related gene (TNFα) in the liver were significantly upregulated in ducks treated with AFB_1_ compared to those in the control and TLE groups (Figure 2). Additionally, the mRNA expression levels of CYP1A1 and CYP1A2 in the liver were increased in the AFB_1_ group compared with those of the control group.

## 3. Discussion

### 3.1. Antioxidative Capacity of T. laurifolia Extract

The *T. laurifolia* extract is a traditional Thai herbal medication known for its antioxidative capacity [33]. One of the main active ingredients of TLE is total phenolic compounds. A previous study [34] indicated a positive correlation exists for other antioxidant capacity methods, such as DPPH and FRAP with polyphenols. The present examination not only investigated the antioxidative capacity of TLE by determining the ABTS, DPPH, and FRAP but also tested the active compound phenolic content. The TLE of the current study exhibited lower activities in terms of DPPH, ABTS, and total phenolic compounds compared to another study [35]. While phytobiotics offer various significant benefits for livestock health, their drawback lies in the variability of composition influenced by factors such as harvesting season and geographical location [36]. This variability may also be one of the reasons why a higher concentration (200 mg/kg TLE) was required to have a noticeable AFB_1_ detoxification effect in this trial.

### 3.2. Aflatoxin B_1_ Toxicity on Growth Performance

The regulatory limit for AFB_1_ in the EU, FDA, and China is 0.02 mg/kg for ducklings [37,38,39]. However, this limit level serves as a precautionary measure to prevent the potential harmful accumulation of AFB_1_ in the bodies of animals after long-term ingestion (over four weeks). Previous research has indicated that AFB_1_ concentration can impair duck production, and significant hepatic lesions can occur at levels as low as 0.5 mg/kg for a short period (lower than four weeks) [40,41]. Taking into account the treatment period (five weeks), experimental efficiency, and various national regulations, we compromised and chose 0.1 mg/kg as the tested content. 

It is well established that AFB_1_ can interfere with poultry energy metabolism, reducing growth efficiency [36,37]. Among poultry, meat ducks are susceptible to aflatoxins. A diet containing a high concentration of AFB_1_ can cause acute death in meat-type ducks, while prolonged exposure to low levels of AFB_1_ can induce chronic toxicity, resulting in growth retardation and reduced production [42]. Previous research has indicated that poultry-fed diets containing aflatoxins as low as 0.3 mg/kg started to show reductions in growth rate, and feed intake and feed efficiency worsened [43]. In the current study, the results indicated that a diet containing 0.1 mg/kg of AFB_1_ led to a reduction in ADG and poor FCR in ducklings. Unlike ADG and FCR, the ADFI of ducklings remained unaffected by AFB_1_ toxicity, which aligns with the effects of AFB_1_ on early young broiler research [44]. 

### 3.3. Aflatoxin B_1_ Toxicity on Serum Biochemical Parameters

Hepatotoxicity is the primary characteristic of AFB_1_ toxicity in numerous animal species [5]. Blood AST, ALT, and alkaline phosphatase (ALP) levels are commonly used as indicators when measuring the effects of aflatoxin on liver toxicity in poultry [45]. Globulin involves several physiological processes, including lipid transportation in birds [15]. Our study revealed that AFB_1_ altered serum biochemical parameters, leading to significantly higher levels of total cholesterol, triglycerides, AST, and globulin. However, the levels of ALT and ALP in the AFB_1_ group did not show a significant increase compared to the control group in our study. This may be attributed to the AFB_1_ concentration in this research not reaching the toxic level required for severe liver damage, which would release high amounts of ALT and ALP. The results of the relative liver weight in our experiment support this. Although the liver weights of the AFB_1_ group were heavier than those of the control and other treatment groups, the difference was not statistically significant. Similar results were observed in other experiments. For instance, adding over 0.5 mg/kg of AFB_1_ to broiler diets can increase serum ALP, ALT, and AST activities [46]. However, when the dietary AFB_1_ concentration was lower than 0.03 mg/kg, only serum AST levels were significantly increased in broilers [5]. The AFB_1_-induced increase in serum total cholesterol and triglycerides observed in this study is consistent with previous research findings [47,48]. The liver plays a crucial role in blood fatty acid metabolism [49], while AFB_1_ induces liver damage and can lead to abnormal triglyceride metabolism.

### 3.4. Aflatoxin B_1_ Toxicity on Intestine Morphology

Aflatoxin B_1_ can alter intestinal morphology, leading to reduced nutrient absorption and subsequent growth retardation [50,51]. However, the effects of AFB_1_ toxicities on poultry intestinal morphology are not entirely clear. This lack of clarity may stem from differences in the specific sections of the intestine, tested variables, and exposure time in previous studies [46]. Additionally, the species and age of poultry used in various studies may also play crucial roles in the intestine’s response to chronic aflatoxicosis. An earlier study indicated that AFB_1_ can induce morphological alterations of the intestinal epithelium by increasing the depth of the crypts, particularly in the small intestine (duodenum and jejunum) [52]. While these findings were consistent with the observations in the duodenum and jejunum, they did not align with those of the ileum in the present study. Furthermore, most research has indicated that AFB_1_ decreased VH in the small intestine of broilers. However, contrary to the observations in broilers [46,52], AFB_1_ had no effect on VH in laying hens [53]. The results of our meat duckling trial also differed from those of the broiler chicken test. Surprisingly, the VH of the duodenum, jejunum, and ileum were all significantly increased by AFB_1_ toxicity. Alterations in both the height and width of villi were also noted in ducks treated with AFB_1_. The alterations in the structure of villi were a result of the activation of the apoptotic pathway by AFB_1_, which subsequently may be related to the absorption of nutrients. Given the differences in these results of intestinal morphology, in addition to the abovementioned differences in varieties and sampling locations, further testing may be necessary to verify and confirm these findings.

### 3.5. Aflatoxin B_1_ Toxicity on Carcass Traits and Meat Quality

Several interesting results were observed regarding carcass traits and meat quality. In contrast to other reports [5,10], our results did not show significant changes in the relative weights of the liver and other organs. Although there was a slight increase in the AFB_1_-contaminated treatment group compared with the control group, this difference did not reach statistical significance. This could be attributed to the tested concentrations of AFB_1_ in this study causing mild hepatotoxicity that did not reach the threshold to alter liver weight. In terms of meat quality, it was discovered that the color of the meat in the AFB_1_-contaminated group showed a significant increase. To the best of our knowledge, there were no other poultry reports that investigated whether AFB_1_ changes the color of poultry meat. However, we found a sheep report [54] indicating that AFB_1_ altered the lightness (L value) of the meat but not the yellowness (b value). Although there were slight differences between the results of the former study and ours, these variations may be attributed to differences in animal species. Nonetheless, it is plausible that AFB_1_ could indeed cause changes in meat color. We speculated that disruptions in pigment metabolism and inflammatory responses associated with liver damage could also influence the color of the meat, potentially contributing to changes in its yellowness [55].

### 3.6. Aflatoxin B_1_ Toxicity on Immunity and Cytochrome P450 Enzyme-Related Genes

Aflatoxin B_1_ induces oxidative damage and apoptosis in hepatocyte cells and is primarily metabolized by cytochrome P450 (CYP450) enzymes [56]. In poultry liver, AFB1 is bioactivated by enzymes such as CYP1A1, CYP1A2, and other enzymes (e.g., CYP2A6 and CYP3A4). CYP450 enzymes convert AFB_1_ into an electrophilic, highly reactive, and unstable metabolite known as aflatoxin-8,9-epoxide (AFBO) [57,58]. This metabolite can interact with cellular macromolecules, binding to guanine residues in DNA, causing genotoxicity, and reacting with proteins to induce cytotoxicity [59]. These interactions result in irreversible DNA damage and can lead to hepatocarcinoma in humans, primates, and ducks [60]. Consistent with previous research in broiler chickens [61], our study observed that AFB_1_ exposure led to a significant increase in CYP1A1 and CYP1A2 mRNA expression. Additionally, our findings were consistent with previous studies, which demonstrated that AFB_1_ treatment increased the mRNA levels of TNF-α [62,63]. This indicates that AFB_1_ toxicity induces the immune response and inflammatory cytokine production in ducklings. However, the mRNA expressions of these enzymes and TNF-α were lower in groups treated with TLE and a commercial binder, suggesting that these feed additives effectively neutralize the hepatotoxic effects of AFB_1_.

### 3.7. Antimycotoxigenic Efficacies of Thunbergia laurifolia Lindl.

Aflatoxin B_1_ is primarily metabolized through CYP1A1 and CYP1A2, producing a highly reactive intermediate (AFBO), which induces the formation of reactive oxygen species (ROS) within hepatocytes [64]. The accumulation of ROS leads to oxidative stress, characterized by an imbalanced response between the production of reactive species and the ability of cells to detoxify or repair the damage [65]. Reactive oxygen species damage cellular components, including lipids, proteins, and DNA, initiating lipid peroxidation and compromising membrane integrity, ultimately leading to cell damage and death [66]. Oxidative damage and cellular stress induce a series of inflammatory responses in the liver, further aggravating tissue damage. Liver damage impairs critical functions, such as detoxification, protein synthesis, and nutrient metabolism, leading to reduced nutrient absorption and utilization, which contributes to poor growth performance [67]. 

Therefore, *T. laurifolia* with natural antioxidants may be a promising option to neutralize AFB_1_ toxicity. Much research has indicated that *T. laurifolia* possesses antioxidant and anti-inflammatory properties, as well as anticancer activities, due to its ability to increase catalase (CAT) and glutathione peroxidase (GPx) activities, thereby removing ROS [68,69,70]. Previous research on chickens has shown promising results using 2% *T. laurifolia* leaf [71]. This treatment ameliorated the adverse effects of multiple mycotoxin-contaminated feeds, improving nutrient digestibility and increasing the activity of glutathione peroxidase. However, it did not lead to a significant change in the growth rate. Our research further investigated the potential of TLE in mitigating the effects of AFB_1_ on growth reduction and hepatoxicity. By utilizing extracts of *T. laurifolia* in our study, we hypothesized that some impurities were eliminated to enhance the concentration of bioactive chemicals, such as total phenolic compounds [72]. Therefore, we only used 100 mg/kg TLE to improve the growth reduction caused by AFB_1_, and the treatment of 200 mg/kg TLE had a stronger detoxification ability, as observed in growth performance, serum biochemical traits, intestinal morphology, and meat quality.

Our results suggest that supplementing TLE into duckling diets could be a natural and effective detoxifying agent against AFB_1_ contamination. This can lead to improved growth performance, feed efficiency, and overall health in poultry, which is crucial for the poultry industry. Additionally, the study presented that TLE improves meat quality by mitigating the adverse effects of AFB_1_. This is critical for ensuring that the meat produced is safe and high quality. Our findings pave the way for further research into the use of TLE for detoxifying various mycotoxins in different animal species.

## 4. Conclusions

It can be concluded that dietary supplementation of *T. laurifolia* extract in ducklings ameliorated the adverse effects of AFB_1_ on growth performance, alleviated liver damage by increasing the drug-metabolizing enzymes (Cytochrome P450), and improved the intestinal health of ducks through participation in their detoxification.

## 5. Materials and Methods

### 5.1. Animal and Ethical Approval

A total of 180 seven-day-old Cherry Valley ducks were obtained from the Faculty of Agriculture, Chiang Mai University, Thailand. The ducks were housed in pens with strict biosecurity measures, with each treatment containing 3 replications of 12 birds each. Over the 35-day duration of the experiment, the ducks received water and feed ad libitum (Table 7). All experimental procedures in this study were conducted strictly in accordance with the recommended guidelines and were submitted for ethical approval by the Animal Ethics Committee, Faculty of Agriculture, Chiang Mai University.

### 5.2. Plant Materials

The mature leaves of *T. laurifolia* Lindl. were collected from Hangdong District, Chiang Mai Province, Thailand. The leaves were cleaned, chopped into pieces, and then oven dried at 60 °C for 24 to 48 h. Subsequently, the dried leaves were powdered using a dry grinder to obtain particles of approximately 0.2 mm in size. The powdered material was stored in a light-resistant container until it was used for the extraction studies. 

### 5.3. Extraction Method and Phenolic Content Measurement

The procedure involved soaking the powdered *T. laurifolia* leaves in boiling distilled water (1:10 *w*/*v*) for one hour. Subsequently, the mixture was passed through a filter paper (Whatman No. 41) and three layers of gauze. The filtrate obtained was freeze-dried and kept in a desiccator at a temperature of 4 °C. To facilitate future use, the extract was diluted in distilled water to achieve the appropriate concentrations and then stored at a temperature of −20 °C. The Folin–Ciocalteu technique [73] was employed to quantify the total phenolic content. The extract was combined with the Folin–Ciocalteu reagent and a 7.5% (*w*/*v*) solution of NaCO_3_. The calibration standard for gallic acid was established by incubating it for 60 min and using a UV–Vis spectrophotometer (SPECTROstar Nano, BMG LABTECH, Ortenberg, Germany). The extract’s total phenolic content was determined in milligrams of gallic acid per gram.

### 5.4. Antioxidative Assays

The DPPH and ABTS radical scavenging activities were evaluated using modified methods based on Sunanta et al. [74] and Sangta et al. [44], respectively. For the DPPH assay, 25 µL of the extract was mixed with 250 µL of 0.20 mM DPPH (2,2-diphenyl-1-picrylhydrazyl) solution. The mixture was then incubated at room temperature, in the dark, for 30 min, and the absorbance was measured at 517 nm. Regarding the ABTS assay, 200 µL of the extract was mixed with 500 µL of a working solution containing 7.00 mM ABTS [2,2-azino-bis-(3-ethylbenzothiazoline-6-sulfonic acid)] and 2.45 mM potassium persulfate. The mixture was incubated in the dark at room temperature for 12–16 h, and the absorbance of the samples was measured at 734 nm. The FRAP was determined using the modified Aljadai method [75]. In this method, 10 µL of the extract was mixed with 190 µL of FRAP reagent for 30 min in the dark, and the absorbance was measured at 593 nm using ascorbic acid as a standard reference.

### 5.5. Treatment Diet Preparation

The powder of AFB_1_ standard (purity ≥ 98%) and commercial binder (Mycosorb Advance) were purchased from Sigma (Saint Louis, MO, USA) and American Colloid Company (Lovell, WY, USA), respectively. One milligram of AFB_1_ standard was dissolved in 100 mL of 95% ethanol (Merck, Darmstadt, Germany) to obtain 10 mg/kg AFB_1_ stock solutions. The prepared solution was then sprayed evenly on the basal feed and mixed to obtain the 0.1 mg/kg AFB_1_-contaminated diet [76,77]. The equivalent amount of ethanol without AFB_1_ solution was sprayed evenly on the basal feed to obtain the control diet. The treatment concentration of TLE and the commercial binder were calculated, respectively, added uniformly to the diet, and mixed evenly. Mycotoxins were detected in the basal diet using ELISA kits (R-Biopharm, Darmstadt, Germany). The analysis revealed that the quantities present in the sample were as follows: 0.012 mg/kg AFB_1_*,* 0.0212 mg/kg T-2 toxin, 0.015 mg/kg ochratoxin A, 0.035 mg/kg zearalenone, and 0.015 mg/kg deoxynivalenol, respectively. 

### 5.6. Growth Performance

All ducklings were fed treatment diets for 35 days. The ducks were clinically observed at least twice daily, and mortality was recorded. Furthermore, the ducks were individually weighed on the age of day 7 and day 42. The performance variables measured in this study include BW, ADG, ADFI, and FCR.

### 5.7. Blood Characteristics

Blood samples were collected at day 42 from each treatment (6 birds) for biochemical analyses. The blood samples were then centrifuged at 3000× *g* for 15 min, and the serum was separated to determine liver function parameters such as AST, ALT, ALP, total protein, globulin, and albumin. All blood characteristics were measured using a BioMajesty^®^ JCA-BM6010/C kit from DiaSys Diagnostic Systems (Holzheim, Germany) with an automated chemistry analyzer BX-301 (Asia Green, Singapore).

### 5.8. Relative Organ Weight

Following the bleeding process, all ducks from each treatment were euthanized via cervical dislocation. Subsequently, the liver, kidney, heart, spleen, gizzard, and bursa of Fabricius were removed, and their weights were measured. The organs were weighed, and their weights were represented as relative organ weights:

Relative weight = (Organ weight)/(Final BW) × 100.

### 5.9. Carcass and Meat Quality

After 42 days of testing, each duck was carefully weighed before being exsanguinated and sacrificed via cervical dislocation. The weight of the carcass (excluding the neck and feet), breast meat, liver, gizzard, pancreas, thymus, bursa of Fabricius, spleen, and abdominal fat was extracted and measured after being rinsed with saline solution. Organ size was quantified as a proportion of BW. The pH of the breast meat was determined using a calibrated glass-electrode pH meter (WTW pH 340-A, WTH Measurement Systems Inc., Ft. Myers, FL). The lightness (L*), redness (a*), and yellowness (b*) values of the breast meat were measured using a Minolta CR410 Chromameter from Konica Minolta Sensing Inc., located in Osaka, Japan. The water-holding capacity (WHC) was determined following the procedures outlined by Kauffman et al. [78]. Additionally, the drip loss was quantified using roughly 2 g of heated material, following the plastic bag technique outlined by Honikel [79]. Subsequently, the cooking loss was calculated using the methodology laid out by Sullivan et al. [80]. The TBARS were quantified using the technique outlined by Witte et al. [81], with the results expressed as milligrams of MDA per kilogram of muscle. The extraction process involved the use of a solution of trichloroacetic acid with a concentration of 20% by weight/volume.

### 5.10. Immune Response and Metabolizing Cytochrome P450 Enzyme-Related Genes Expression in the Liver

At the end of the experiment, three birds were randomly selected from each treatment, and their liver tissues were immediately removed and frozen at −80 °C until RNA extraction. Total RNA was extracted from 50 mg of liver samples homogenized with liquid nitrogen using Trizol and a columnar RNA extraction kit (Invitrogen, PureLink^TM^ RNA Mini Kit, Thermo Scientific, Wilmington, NC, USA) according to the manufacturer’s protocol. The extracted RNA was quantified using a spectrophotometer (NanoDropTM 2000, Thermo Scientific, Wilmington, NC, USA) at an absorbance ratio of 260–280 nm. Subsequently, the cDNA was synthesized using a cDNA synthesis kit (iScriptTM cDNA Synthesis Kit, BIO-RAD, Hercules, CA, USA) according to the manufacturer’s instructions.

The qPCR reaction was carried out using the CFX ConnectTM Real-Time PCR System (BIO-RAD, Hercules, CA, USA) with the iTaq Universal SYBR Green supermix 2X (BIO-RAD, Hercules, CA, USA) and specific primers for individual genes (Table 8). Changes in the expression levels of the above genes were measured using the 2-ΔΔCt method and a standard curve, as outlined by Larionov et al. [82].

### 5.11. Statistical Analysis 

The experimental data were analyzed using the analysis of variance (ANOVA) procedure of SAS Enterprise Guide Software V.9.4 (SAS Institute, Cary, NC, USA). The least square means (LSM) were compared using Tukey’s test, and a probability level of *p* < 0.05 was considered statistically significant.

## Figures and Tables

**Table 1:** Total phenolic compounds and antioxidant activity of *T. laurifolia* extract.

	Total Phenol Compound (mg GAE/g)	DPPH (μmol TE/g)	^1^ABTS (IC_50_)	FRAP (mM Fe^2+/^g)
*T. laurifolia* extract	0.56 ± 0.05	7.26 ± 0.98	3.70 ± 0.52	51.26 ± 1.5

^1^ABTS: 2,2-azino-bis (3-ethylbenzothiazo-line-6-sulfonic acid); DPPH: 2,2-diphenyl-1-picrylhydrazyl radical; FRAP: ferric reducing antioxidant power; Fe^2+^: iron divalent ions; GAE: gallic acid equivalents; IC_50_: half maximal inhibitory concentration; TE: Trolox equivalents.

**Table 2:** Effects of *T. laurifolia* extract on growth parameters of aflatoxin B_1_-challenged ducklings.

Item	^1^T1	T2	T3	T4	T5	SEM	*p*-Value
Initial BW, g	88.3	82.8	84.7	82.7	86.0	1.61	0.1290
Final BW, g	2303.0 ^a^	1831.4 ^b^	2244.9 ^a^	2307.3 ^a^	2236.6 ^a^	44.06	<0.0001
ADG, g	63.3 ^a^	50.0 ^b^	61.7 ^a^	63.6 ^a^	61.4 ^a^	1.82	<0.0001
ADFI, g	145.8	144.9	144.1	139.9	139.7	1.27	0.0880
FCR	2.3 ^b^	2.9 ^a^	2.3 ^b^	2.2 ^b^	2.3 ^b^	0.08	0.0001

^a–b^ Means with different superscripts in a column differ significantly (*p* < 0.05). ^1^T1: Control, only basal diet without AFB_1_, TLE or commercial binder; T2: the basal diet containing 0.1 mg AFB_1_/kg; T3: the basal diet containing 0.1 mg AFB_1_/kg and 100 mg TLE/kg; T4: the basal diet containing 0.1 mg AFB_1_/kg and 200 mg TLE/kg; T5: the basal diet containing 0.1 mg AFB_1_/kg and 0.5 g/kg of commercial binder; AFB_1_: aflatoxin B_1_; TLE: *T. laurifolia* extract; BW, body weight; ADG, average daily gain; ADFI, average daily feed intake; FCR, feed conversion ratio.

**Table 3:** Effects of *T. laurifolia* extract on serum biochemical of aflatoxin B_1_-challenged ducklings.

Item	^1^T1	T2	T3	T4	T5	SEM	*p*-Value
Total cholesterol, mg/dL	110.0 ^c^	151.0 ^ab^	161.3 ^a^	157.7 ^ab^	129.3 ^bc^	9.28	0.0135
Triglyceride, mg/dL	122.0 ^c^	183.3 ^ab^	177.0 ^ab^	158.0 ^bc^	218.3 ^a^	16.51	0.0232
AST, U/L	27.0 ^d^	43.3 ^a^	33.0 ^c^	29.7 ^cd^	37.7 ^b^	1.41	<0.0001
ALT, U/L	32.7 ^ab^	62.3 ^a^	47.3 ^a^	32.3 ^b^	44.0 ^a^	3.17	0.0001
ALP, U/L	846.3 ^a^	834.0 ^a^	749.7 ^b^	662.0 ^c^	833.67 ^a^	18.05	0.1922
Total protein, mg/dL	2.73 ^c^	2.87 ^bc^	3.03 ^abc^	3.30 ^a^	3.23 ^ab^	0.12	0.0431
Albumin, mg/dL	1.37	1.37	1.27	1.43	1.43	0.06	0.2742
Globulin, mg/dL	1.37 ^b^	1.67 ^a^	1.60 ^ab^	1.86 ^a^	1.80 ^a^	0.08	0.0118

^a–d^ Means with different superscripts in a column differ significantly (*p* < 0.05). ^1^T1: Control, only basal diet without AFB_1_, TLE or commercial binder; T2: the basal diet containing 0.1 mg AFB_1_/kg; T3: the basal diet containing 0.1 mg AFB_1_/kg and 100 mg TLE/kg; T4: the basal diet containing 0.1 mg AFB_1_/kg and 200 mg TLE/kg; T5: the basal diet containing 0.1 mg AFB_1_/kg and 0.5 g/kg of commercial binder; AFB_1_: aflatoxin B_1_; TLE: *T. laurifolia* extract; AST: aspartate transaminase; ALT: alanine transaminase; ALP: alkaline phosphatase.

**Table 4:** Effects of *T. laurifolia* extract supplementation on intestinal morphology of aflatoxin B_1_-challenged ducklings.

Item	^1^T1	T2	T3	T4	T5	SEM	*p*-Value
Duodenum							
VH	904.36 ^e^	974.41 ^d^	989.81 ^c^	1069.15 ^b^	1098.01 ^a^	1.32	<0.0001
VW	115.85 ^d^	133.42 ^ab^	136.02 ^a^	126.65 ^c^	128.02 ^bc^	1.08	0.0015
CD	214.37 ^b^	222.21 ^a^	226.21 ^a^	178.33 ^c^	166.74 ^d^	0.96	<0.0001
VH:CD	4.52 ^c^	4.62 ^c^	4.75 ^c^	6.58 ^b^	7.31 ^a^	0.10	<0.0001
Jejunum							
VH	775.95 ^e^	782.95 ^d^	799.55 ^c^	857.01 ^b^	961.79 ^a^	1.15	<0.0001
VW	113.55 ^ab^	103.52 ^c^	109.12 ^b^	117.88 ^a^	117.59 ^a^	0.96	0.0028
CD	174.95 ^b^	181.74 ^a^	180.64 ^a^	167.56 ^c^	150.76 ^d^	0.97	<0.0001
VH:CD	4.56 ^c^	4.53 ^c^	4.37 ^c^	5.78 ^b^	6.57 ^a^	0.05	<0.0001
Ileum							
VH	539.73 ^e^	616.03 ^d^	627.63 ^c^	700.65 ^a^	650.57 ^b^	1.76	<0.0001
VW	98.39	93.37	98.77	94.85	91.33	1.67	0.2557
CD	101.35 ^a^	86.01 ^b^	90.03 ^b^	102.74 ^a^	84.81 ^b^	1.11	0.0011
VH:CD	6.00 ^d^	7.33 ^c^	7.93 ^ab^	7.56 ^bc^	8.18 ^a^	0.11	0.0013

^a–e^ Means with different superscripts in a column differ significantly (*p* < 0.05). ^1^T1: Control, only basal diet without AFB_1_, TLE or commercial binder; T2: the basal diet containing 0.1 mg AFB_1_/kg; T3: the basal diet containing 0.1 mg AFB_1_/kg and 100 mg TLE/kg; T4: the basal diet containing 0.1 mg AFB_1_/kg and 200 mg TLE/kg; T5: the basal diet containing 0.1 mg AFB_1_/kg and 0.5 g/kg of commercial binder; AFB_1_: aflatoxin B_1_; TLE: *T. laurifolia* extract; VH: villus height; VW: villus width; CD: crypt depth; VH:CD: villus height per crypt depth ratio.

**Table 5:** Effects of *T. laurifolia* extract on carcass trait and relative organ weight of aflatoxin B_1_-challenged ducklings.

Item	^1^T1	T2	T3	T4	T5	SEM	*p*-Value
Carcass traits							
Dressing, %	87.73	86.23	87.30	87.65	85.90	0.57	0.0822
Breast, g	10.81	9.75	10.02	10.48	11.20	0.52	0.2814
Thigh, g	7.49	7.71	7.90	8.22	7.82	0.29	0.4990
Wing, g	12.07	12.07	12.19	12.40	12.56	0.22	0.4555
Relative organ weight, g							
Liver, g	2.24	2.34	2.28	2.04	2.17	0.08	0.0696
Spleen	0.11	0.09	0.09	0.09	0.09	0.01	0.5229
Kidney	0.70	0.74	0.75	0.76	0.72	0.02	0.4222
Bursa of fabricius	0.17	0.18	0.16	0.18	0.17	0.01	0.7154
Heart	0.61	0.65	0.63	0.66	0.65	0.02	0.6874
Gizzard	4.71	4.75	4.79	4.49	5.07	0.14	0.0918

^1^T1: Control, only basal diet without AFB_1_, TLE or commercial binder; T2: the basal diet containing 0.1 mg AFB_1_/kg; T3: the basal diet containing 0.1 mg AFB_1_/kg and 100 mg TLE/kg; T4: the basal diet containing 0.1 mg AFB_1_/kg and 200 mg TLE/kg; T5: the basal diet containing 0.1 mg AFB_1_/kg and 0.5 g/kg of commercial binder; AFB_1_: aflatoxin B_1_; TLE: *T. laurifolia* extract.

**Table 6:** Effect of *T. laurifolia* extract on meat quality of aflatoxin B_1_-challenged ducklings.

Item	^1^T1	T2	T3	T4	T5	SEM	*p*-Value
pH value_45 min_	5.91	6.16	6.21	5.95	5.90	0.12	0.2494
pH value_24 h_	5.60	5.58	5.66	5.57	5.55	0.06	0.6975
Cook loss, %	30.60	31.81	32.52	33.91	33.95	1.30	0.3258
Drip loss, %	2.83	2.20	2.56	2.42	2.48	0.66	0.9743
Shear force, N	32.20 ^b^	34.20 ^b^	29.48 ^b^	32.37 ^b^	45.44 ^a^	3.35	0.0374
TBARS, mg MDA/kg	1.59	1.45	1.50	1.55	1.54	0.06	0.5031
Meat color							
L*	40.50	42.20	43.00	38.80	42.15	1.74	0.4822
a*	15.90	16.79	16.28	14.83	16.71	0.64	0.2671
b*	4.03 ^c^	7.23 ^a^	7.04 ^a^	4.36 ^bc^	6.46 ^ab^	0.72	0.0258

^a–c^ Means with different superscripts in a column differ significantly (*p* < 0.05). ^1^T1: Control, only basal diet without AFB_1_, TLE or commercial binder; T2: the basal diet containing 0.1 mg AFB_1_/kg; T3: the basal diet containing 0.1 mg AFB_1_/kg and 100 mg TLE/kg; T4: the basal diet containing 0.1 mg AFB_1_/kg and 200 mg TLE/kg; T5: the basal diet containing 0.1 mg AFB_1_/kg and 0.5 g/kg of commercial binder; AFB_1_: aflatoxin B_1_; TLE: *T. laurifolia* extract; TBARS: thiobarbituric acid reactive substances; MDA: malondialdehyde; L*: lightness; a*: redness; b*: yellowness.

**Table 7:** The formulation and proximate composition of the experimental diet (g/kg).

Items	1–3 Weeks	4–5 Weeks
Ingredient (g/kg feed)		
Corn meal	700.00	575.00
Rice bran	0.00	75.00
Full-fat soybean meal	0.00	25.00
Soybean meal, 44%	205.00	192.50
Meat meal, 50%	25.00	25.00
Limestone	10.00	25.00
Calcium carbonate	0.00	47.40
Monopotassium phosphate, 22%	10.50	17.50
^1^ Premix	2.50	2.50
Methionine	0.90	1.50
Toxin binder	1.00	0.50
Salt	0.00	2.00
Multi protein plus, 68%	45.00	11.00
Phytase	0.10	0.10
Total	1000.00	1000.00
Nutrient composition (% dry matter basis)		
Moisture	12.23	9.78
Ash	6.79	11.91
Crude protein	22.22	18.0
Crude fiber	4.56	3.82
Crude fat	5.15	4.59
Gross energy (Cal/g)	2964.92	3581.65

^1^ Vitamin premix (per kg premix): vitamin A 19,000,000 IU, vitamin D3 3,900,000 IU, vitamin E 11,500 IU, vitamin K_3_ 4.30 g, vitamin B_1_ 5.50 g, vitamin B_2_ 10.50 g, vitamin B_6_ 4.80 g, vitamin B_12_ 0.19 g, vitamin C 15.50 g, pantothenic acid 15.10 g, folic acid 2.90 g, nicotinic acid 39.00 g, biotin 0.25 g. 2 Mineral premix (per kg premix): magnesium 105.00 g, potassium 89.00 g, sodium 105.00 g, and feed additive 24.50 g.

**Table 8:** Primer sequences, amplicons, and the related information for quantitative real-time PCR.

Target Gene		Primer Sequences	Product Size (bp)
Housekeeping gene			
GAPDH	Forward	CTGGCATTGCACTGAACGAC	165
	Reverse	CTCCAACAAAGGGTCCTGCT	
Immune-related genes			
IL-6	Forward	GCGGAACCAAGAGCAGAGATGAG	130
	Reverse	CCACGGCAGGACTGGATAATAACC	
IL-8	Forward	GCTGTCCTGGCTCTTCTCCT	120
	Reverse	GCACACCTCTCTGTTGTCCTTC	
TNF-α	Forward	CCGTGGTCAGTTTCCATCAGG	117
	Reverse	ACTTTGCAGTTAGGTGACGCT	
P450 (Metabolism of AFB1) genes			
CYP1A1	Forward	AGGACGGAGGCTGACAAGGTG	104
	Reverse	AGGATGGTGGTGAGGAAGAGGAAG	
CYP1A2	Forward	CCACGCAGATCCCAAACGAG	120
	Reverse	TGTGAGGGTACGTCACGAGG	

IL6 = interleukin 6; IL8 = interleukin 8; TNF-α = tumor necrosis factor alpha; CYP1A1 = cytochrome P450 1A1; CYP1A2 = cytochrome P450 1A2.

## Data Availability

Data are contained in the article.

## References

[1] Kumar A., Pathak H., Bhadauria S., Sudan J. (2021). Aflatoxin contamination in food crops: Causes, detection, and management: A review. Food Prod. Process. Nutr..

[2] Awuchi C.G., Ondari E.N., Ogbonna C.U., Upadhyay A.K., Baran K., Okpala C.O.R., Korzeniowska M., Guine R.P.F. (2021). Mycotoxins Affecting Animals, Foods, Humans, and Plants: Types, Occurrence, Toxicities, Action Mechanisms, Prevention, and Detoxification Strategies—A Revisit. Foods.

[3] Qureshi H., Hamid S.S., Ali S.S., Anwar J., Siddiqui A.A., Khan N.A. (2015). Cytotoxic effects of aflatoxin B1 on human brain microvascular endothelial cells of the blood-brain barrier. Med. Mycol..

[4] Pleadin J., Kovacevic D., Perkovic I. (2015). Impact of casing damaging on aflatoxin B1 concentration during the ripening of dry-fermented meat sausages. J. Immunoass. Immunochem..

[5] Zou Y., Liu S.B., Zhang Q., Tan H.Z. (2023). Effects of Aflatoxin B(1) on growth performance, carcass traits, organ index, blood biochemistry and oxidative status in Chinese yellow chickens. J. Vet. Med. Sci..

[6] Kananub S., Jala P., Laopiem S., Boonsoongnern A., Sanguankiat A. (2021). Mycotoxin profiles of animal feeds in the central part of Thailand: 2015-2020. Vet. World.

[7] Waenlor W., Wiwanitkit V. (2003). Aflatoxin contamination of food and food products in Thailand: An overview. Southeast. Asian J. Trop. Med. Public. Health.

[8] Pickova D., Ostry V., Toman J., Malir F. (2021). Aflatoxins: History, Significant Milestones, Recent Data on Their Toxicity and Ways to Mitigation. Toxins.

[9] Alameri M.M., Kong A.S., Aljaafari M.N., Ali H.A., Eid K., Sallagi M.A., Cheng W.H., Abushelaibi A., Lim S.E., Loh J.Y. (2023). Aflatoxin Contamination: An Overview on Health Issues, Detection and Management Strategies. Toxins.

[10] Mesgar A., Aghdam Shahryar H., Bailey C.A., Ebrahimnezhad Y., Mohan A. (2022). Effect of Dietary L-Threonine and Toxin Binder on Performance, Blood Parameters, and Immune Response of Broilers Exposed to Aflatoxin B(1). Toxins.

[11] Tansakul N., Rattanasrisomporn J., Roytrakul S. (2019). Proteomics analysis of serum protein patterns in duck during aflatoxin B1 exposure. Vet. World.

[12] Barraud L., Guerret S., Chevallier M., Borel C., Jamard C., Trepo C., Wild C.P., Cova L. (1999). Enhanced duck hepatitis B virus gene expression following aflatoxin B1 exposure. Hepatology.

[13] Maldjian A., Cristofori C., Noble R.C., Speake B.K. (1996). The fatty acid composition of brain phospholipids from chicken and duck embryos. Comp. Biochem. Physiol. B Biochem. Mol. Biol..

[14] Eraslan G., Essiz D., Akdogan M., Sahindokuyucu F., Altintas L., Hismiogullari S. (2005). Effects of dietary aflatoxin and sodium bentonite on some hormones in broiler chickens. Bull. Vet. Inst. Pulawy.

[15] Tso K.H., Lumsangkul C., Cheng M.C., Ju J.C., Fan Y.K., Chiang H.I. (2021). Differential Effects of Green Tea Powders on the Protection of Brown Tsaiya and Kaiya Ducklings against Trichothecene T-2 Toxin Toxicity. Animals.

[16] Peles F., Sipos P., Kovacs S., Gyori Z., Pocsi I., Pusztahelyi T. (2021). Biological Control and Mitigation of Aflatoxin Contamination in Commodities. Toxins.

[17] Peng Z., Chen L., Zhu Y., Huang Y., Hu X., Wu Q., Nüssler A.K., Liu L., Yang W. (2018). Current major degradation methods for aflatoxins: A review. Trends Food Sci. Technol..

[18] Lai Y., Sun M., He Y., Lei J., Han Y., Wu Y., Bai D., Guo Y., Zhang B. (2022). Mycotoxins binder supplementation alleviates aflatoxin B(1) toxic effects on the immune response and intestinal barrier function in broilers. Poult. Sci..

[19] Chung T.K., Erdman J.W., Baker D.H. (1990). Hydrated sodium calcium aluminosilicate: Effects on zinc, manganese, vitamin A, and riboflavin utilization. Poult. Sci..

[20] Nielsen F.H. (2012). History of zinc in agriculture. Adv. Nutr..

[21] Yesuf Y.K., Tamir B., Tesfaye E., Beyero N. (2023). The synergetic effects of some phytobiotics mix on growth, hematology and microbial loads of broiler chickens. Anim. Biotechnol..

[22] Kikusato M. (2021). Phytobiotics to improve health and production of broiler chickens: Functions beyond the antioxidant activity. Anim. Biosci..

[23] Khunkitti W., Taweechaisupapong S., Aromdee A., Pese M. Antimicrobial activity of *Thunbergia laurifolia* crude extract. Proceedings of the 3rd World Congress on Medicinal Plant and Aromatic Plants for Human Welfare.

[24] Srida C., Hankete J., Aromdee C., Pese M. (2002). Antioxidant activity of *Thunbergia laurifolia* ethanolic extract. Thai J. Pharm. Sci..

[25] Ussanawarong S., Thesiri T. (2001). Effect of *Thunbergia laurifolia* Linn. on detoxication of parathion in rat. Khon Kaen Univ. Res. J..

[26] Tejasen P., Thongthapp C. (1980). The study of the insecticide antitoxicity of *Thunbergia laurifolia* Linn. Chiang Mai Med. Bull..

[27] Palipoch S., Jiraungkoorskul W., Tansatit T., Preyavichyapugdee N., Jaikua W., Kosai P. (2011). Protective efficiency of *Thunbergia laurifolia* leaf extract against lead (II) nitrate-induced toxicity in Oreochromis niloticus. J. Med. Plant Res..

[28] Wonkchalee O., Boonmars T., Aromdee C., Laummaunwai P., Khunkitti W., Vaeteewoottacharn K., Sriraj P., Aukkanimart R., Loilome W., Chamgramol Y. (2012). Anti-inflammatory, antioxidant and hepatoprotective effects of *Thunbergia laurifolia* Linn. on experimental opisthorchiasis. Parasitol. Res..

[29] Oonsivilai R., Cheng C., Bomser J., Ferruzzi M.G., Ningsanond S. (2007). Phytochemical profiling and phase II enzyme-inducing properties of *Thunbergia laurifolia* Lindl. (RC) extracts. J. Ethnopharmacol..

[30] Chan E., Lim Y.Y. (2006). Antioxidant activity of *Thunbergia laurifolia* tea. J. Trop. For. Sci..

[31] Ruela-de-Sousa R.R., Fuhler G.M., Blom N., Ferreira C.V., Aoyama H., Peppelenbosch M.P. (2010). Cytotoxicity of apigenin on leukemia cell lines: Implications for prevention and therapy. Cell Death Dis..

[32] Marin D.E., Taranu I. (2012). Overview on aflatoxins and oxidative stress. Toxin Rev..

[33] Junsi M., Siripongvutikorn S. (2016). *Thunbergia laurifolia*, a traditional herbal tea of Thailand: Botanical, chemical composition, biological properties and processing influence. Int. Food Res. J..

[34] Forester S.C., Lambert J.D. (2011). The role of antioxidant versus pro-oxidant effects of green tea polyphenols in cancer prevention. Mol. Nutr. Food Res..

[35] Essiedu J.A., Gonu H., Adadi P., Usansa U. (2023). Polyphenols and Antioxidant Activity of *Thunbergia laurifolia* Infused Tea under Drying Conditions. J. Food Qual..

[36] Jayasundara N., Arampath P. (2021). Effect of variety, location & maturity stage at harvesting, on essential oil chemical composition, and weight yield of Zingiber officinale roscoe grown in Sri Lanka. Heliyon.

[37] No E. (1881). Commission regulation (EC) No. 1881/2006 of 19 December 2006. Setting maximum levels for certain contaminants in foodstuffs (Text with EEA relevance). Off. J. Eur. Comm..

[38] Ksenija N. (2018). Mycotoxins–climate impact and steps to prevention based on prediction. Acta Vet..

[39] Selvaraj J.N., Wang Y., Zhou L., Zhao Y., Xing F., Dai X., Liu Y. (2015). Recent mycotoxin survey data and advanced mycotoxin detection techniques reported from China: A review. Food Addit. Contam. Part. A Chem. Anal. Control Expo. Risk Assess..

[40] Monson M., Coulombe R., Reed K. (2015). Aflatoxicosis: Lessons from Toxicity and Responses to Aflatoxin B1 in Poultry. Agriculture.

[41] Muller R.D., Carlson C.W., Semeniuk G., Harshfield G.S. (1970). The response of chicks, ducklings, goslings, pheasants and poults to graded levels of aflatoxins. Poult. Sci..

[42] Xie Q., Sun M., Chang W., Liu Z., Ma J., Liu G., Cai H., Wang J., Lyu C. (2015). Effects of aflatoxins and absorbents on growth performance and immune indices of meat ducks. Chin. J. Anim. Nutr..

[43] Chen X., Grenier B. (2013). Aflatoxins in Poultry.

[44] Randall G.M., Bird F.H. (1979). The effect of exercise on the toxicity of aflatoxin B1 in chickens. Poult. Sci..

[45] Malekinezhad P., Ellestad L.E., Afzali N., Farhangfar S.H., Omidi A., Mohammadi A. (2021). Evaluation of berberine efficacy in reducing the effects of aflatoxin B1 and ochratoxin A added to male broiler rations. Poult. Sci..

[46] Yunus A.W., Razzazi-Fazeli E., Bohm J. (2011). Aflatoxin B(1) in affecting broiler’s performance, immunity, and gastrointestinal tract: A review of history and contemporary issues. Toxins.

[47] Rotimi O.A., Rotimi S.O., Duru C.U., Ebebeinwe O.J., Abiodun A.O., Oyeniyi B.O., Faduyile F.A. (2017). Acute aflatoxin B1—Induced hepatotoxicity alters gene expression and disrupts lipid and lipoprotein metabolism in rats. Toxicol. Rep..

[48] El-Nekeety A.A., Abdel-Azeim S.H., Hassan A.M., Hassan N.S., Aly S.E., Abdel-Wahhab M.A. (2014). Quercetin inhibits the cytotoxicity and oxidative stress in liver of rats fed aflatoxin-contaminated diet. Toxicol. Rep..

[49] Alves-Bezerra M., Cohen D.E. (2017). Triglyceride metabolism in the liver. Compr. Physiol..

[50] Han X.-Y., Huang Q.-C., Li W.-F., Jiang J.-F., Xu Z.-R. (2008). Changes in growth performance, digestive enzyme activities and nutrient digestibility of cherry valley ducks in response to aflatoxin B1 levels. Livest. Sci..

[51] Zhang M., Li Q., Wang J., Sun J., Xiang Y., Jin X. (2022). Aflatoxin B1 disrupts the intestinal barrier integrity by reducing junction protein and promoting apoptosis in pigs and mice. Ecotoxicol. Environ. Saf..

[52] Poloni V., Magnoli A., Fochesato A., Cristofolini A., Caverzan M., Merkis C., Montenegro M., Cavaglieri L. (2020). A Saccharomyces cerevisiae RC016-based feed additive reduces liver toxicity, residual aflatoxin B1 levels and positively influences intestinal morphology in broiler chickens fed chronic aflatoxin B1-contaminated diets. Anim. Nutr..

[53] Applegate T.J., Schatzmayr G., Prickel K., Troche C., Jiang Z. (2009). Effect of aflatoxin culture on intestinal function and nutrient loss in laying hens. Poult. Sci..

[54] Cao Q.Q., Lin L.X., Xu T.T., Lu Y., Zhang C.D., Yue K., Huang S.C., Dong H.J., Jian F.C. (2021). Aflatoxin B1 alters meat quality associated with oxidative stress, inflammation, and gut-microbiota in sheep. Ecotoxicol. Environ. Saf..

[55] Seideman S.C., Cross H.R., Smith G.C., Durland P.R. (1984). Factors associated with fresh meat color: A review. J. Food Qual..

[56] Diaz G.J., Murcia H.W., Cepeda S.M. (2010). Cytochrome P450 enzymes involved in the metabolism of aflatoxin B1 in chickens and quail. Poult. Sci..

[57] Do J.H., Choi D.-K. (2007). Aflatoxins: Detection, toxicity, and biosynthesis. Biotechnol. Bioprocess. Eng..

[58] Stern A., Furlan V., Novak M., Stampar M., Kolenc Z., Kores K., Filipic M., Bren U., Zegura B. (2021). Chemoprotective Effects of Xanthohumol against the Carcinogenic Mycotoxin Aflatoxin B1. Foods.

[59] Ferguson L.R., Philpott M. (2008). Nutrition and mutagenesis. Annu. Rev. Nutr..

[60] Diaz G.J., Murcia H.W., Cepeda S.M., Boermans H.J. (2010). The role of selected cytochrome P450 enzymes on the bioactivation of aflatoxin B1 by duck liver microsomes. Avian Pathol..

[61] Liu W.C., Yang Y.Y., Pushparaj K., Balasubramanian B. (2022). Evaluation of Hepatic Detoxification Effects of Enteromorpha prolifera Polysaccharides against Aflatoxin B(1) in Broiler Chickens. Antioxidants.

[62] Hou L., Qiu H., Li A., Dong J., Zhu L., Liu G., Chen F. (2022). Effects of aflatoxin B(1) on growth performance, antioxidant status, immune response, and pro-inflammatory cytokine mRNA expression in ISA chicks. Front. Vet. Sci..

[63] Li Y., Ma Q.G., Zhao L.H., Wei H., Duan G.X., Zhang J.Y., Ji C. (2014). Effects of lipoic acid on immune function, the antioxidant defense system, and inflammation-related genes expression of broiler chickens fed aflatoxin contaminated diets. Int. J. Mol. Sci..

[64] Benkerroum N. (2020). Chronic and Acute Toxicities of Aflatoxins: Mechanisms of Action. Int. J. Environ. Res. Public Health.

[65] Pizzino G., Irrera N., Cucinotta M., Pallio G., Mannino F., Arcoraci V., Squadrito F., Altavilla D., Bitto A. (2017). Oxidative Stress: Harms and Benefits for Human Health. Oxid. Med. Cell Longev..

[66] Juan C.A., Pérez de la Lastra J.M., Plou F.J., Pérez-Lebeña E. (2021). The Chemistry of Reactive Oxygen Species (ROS) Revisited: Outlining Their Role in Biological Macromolecules (DNA, Lipids and Proteins) and Induced Pathologies. Int. J. Mol. Sci..

[67] Allameh A., Niayesh-Mehr R., Aliarab A., Sebastiani G., Pantopoulos K. (2023). Oxidative Stress in Liver Pathophysiology and Disease. Antioxidants.

[68] Junsi M., Takahashi Yupanqui C., Usawakesmanee W., Slusarenko A., Siripongvutikorn S. (2020). *Thunbergia laurifolia* Leaf Extract Increased Levels of Antioxidant Enzymes and Protected Human Cell-Lines In Vitro Against Cadmium. Antioxidants.

[69] Palipoch S., Punsawad C., Suwannalert P. (2013). *Thunbergia laurifolia*, a new choice of natural antioxidant to prevent oxidative stress-related pathology: A review. J. Med. Plants Res..

[70] Junsi M., Siripongvutikorn S., Yupanqui C., Usawakesmanee W. (2017). Phenolic and flavonoid compounds in aqueous extracts of *Thunbergia laurifolia* leaves and their effect on the toxicity of the carbamate insecticide methomyl to murine macrophage cells. Funct. Foods Health Dis..

[71] Donkotjan C., Benjanirut C., Angkanaporn K. (2020). Effect of *Thunbergia laurifolia* leaves on the growth performance, nutrient digestibility and liver antioxidant enzymes of broilers fed mycotoxin-contaminated feed. Anim. Prod. Sci..

[72] Zhang J., Wen C., Zhang H., Duan Y., Ma H. (2019). Recent advances in the extraction of bioactive compounds with subcritical water: A review. Trends Food Sci. Technol..

[73] Lamuela-Raventós R.M. (2018). Folin–Ciocalteu method for the measurement of total phenolic content and antioxidant capacity. Meas. Antioxid. Act. Capacit. Recent. Trends Appl..

[74] Sunanta P., Chung H.H., Kunasakdakul K., Ruksiriwanich W., Jantrawut P., Hongsibsong S., Sommano S.R. (2020). Genomic relationship and physiochemical properties among raw materials used for Thai black garlic processing. Food Sci. Nutr..

[75] Kek S.P., Chin N.L., Yusof Y.A., Tan S.W., Chua L.S. (2017). Classification of entomological origin of honey based on its physicochemical and antioxidant properties. Int. J. Food Prop..

[76] Li S., Han M., Zhang Y., Ishfaq M., Liu R., Wei G., Zhang X., Zhang X. (2022). Effect of Curcumin as feed supplement on immune response and pathological changes of broilers exposed to Aflatoxin B1. Biomolecules.

[77] Lin M.-J., Chang S.-C., Tso K.-H., Lin W.-C., Chang C.-L., Lee T.-T. (2018). Effect of T-2 toxin and antioxidants on angel wing incidence and severity in White Roman geese. J. Appl. Anim. Res..

[78] Kauffman S.A. (1986). Autocatalytic sets of proteins. J. Theor. Biol..

[79] Honikel K.O. (1998). Reference methods for the assessment of physical characteristics of meat. Meat Sci..

[80] O’sullivan M., Byrne D., Jensen M., Andersen H.J., Vestergaard J. (2003). A comparison of warmed-over flavour in pork by sensory analysis, GC/MS and the electronic nose. Meat Sci..

[81] Witte V.C., Krause G.F., Bailey M.E. (1970). A new extraction method for determining 2-thiobarbituric acid values of pork and beef during storage. J. Food Sci..

[82] Larionov A., Krause A., Miller W. (2005). A standard curve based method for relative real time PCR data processing. BMC Bioinform..

